# Coagulation test understanding and ordering by medical trainees: Novel teaching approach

**DOI:** 10.1002/rth2.12746

**Published:** 2022-06-17

**Authors:** Nadia Gabarin, Martina Trinkaus, Rita Selby, Nicola Goldberg, Hina Hanif, Michelle Sholzberg

**Affiliations:** ^1^ Department of Medicine, Michael G. DeGroote School of Medicine McMaster University Hamilton Ontario Canada; ^2^ Department of Medicine St. Michael’s Hospital University of Toronto Toronto Ontario Canada; ^3^ Division of Hematology, Department of Medicine, St. Michael’s Hospital University of Toronto Toronto Ontario Canada; ^4^ Department of Laboratory Medicine & Pathobiology and Department of Medicine University Health Network and Sunnybrook Health Sciences Centre, University of Toronto Toronto Ontario Canada; ^5^ Department of Laboratory Medicine & Pathobiology St. Michael’s Hospital University of Toronto Toronto Ontario Canada

**Keywords:** coagulation, INR, laboratory, medical students, physicians

## Abstract

**Background:**

Coagulation testing provides a prime opportunity to make an impact on the reduction of unnecessary laboratory test ordering, as there are clear indications for testing. Despite the prothrombin time/international normalized ratio and activated partial thromboplastin time being validated for specific clinical indications, they are frequently ordered as screening tests and often ordered together, suggesting a gap in understanding of coagulation.

**Methods:**

Based on a needs assessment, we developed an online educational module on coagulation for trainees, incorporating education on testing cost, specificity, and sensitivity. Fifty participating resident physicians and medical students completed a validated premodule quiz, postmodule quiz after completion of the module, and a latent quiz 3 to 6 months after to assess longer‐term knowledge retention. Trainees provided responses regarding their subjective laboratory test‐ordering practices before and after module completion.

**Results:**

The median premodule quiz score was 67% (n = 50; range, 24%‐86%) with an increase of 24% to a median postmodule quiz score of 91% (n = 50; range, 64%‐100%). There was evidence of sustained knowledge acquisition with a latent quiz median score of 89% (n = 40; range, 67%–100%). Trainees were more likely to consider the sensitivity, specificity, and cost of laboratory investigations before ordering them following completion of the educational module.

**Conclusions:**

Using the expertise of medical educators and incorporating trainee feedback, we employed a novel approach to the teaching of coagulation to maximize its approachability and clinical relevance. We found sustained knowledge retention regarding coagulation and appropriate coagulation test ordering, and a subjective change to trainee ordering habits following participation in our educational intervention.

**Essentials**

- Inappropriate international normalized ratio (INR)/activated partial thromboplastin time (aPTT) testing in unselected patients remains a problem in the inpatient setting.
- Medical trainees carry out a large proportion of laboratory test ordering in the hospital.
- Our e‐module on INR/aPTT testing enhanced trainee knowledge of coagulation and appropriate testing.
- Innovative and remotely accessible e‐learning resources can bridge the coagulation knowledge gap.

## 1 INTRODUCTION

Choosing Wisely, an initiative launched by the American Board of Internal Medicine Foundation with the goal of reducing unnecessary laboratory investigations and treatments, has led to greater emphasis on resource stewardship in medicine. However, overutilization of laboratory tests continues to be a problem that results in unnecessary costs, a cascade of additional investigations or treatments, and may even result in patient harm.^1^, ^2^, ^3^, ^4^, ^5^

Coagulation testing is a prime opportunity for utilization initiatives, as there are clear indications for testing and harms associated with inappropriate use of these tests. The prothrombin time (PT)/international normalized ratio (INR) was validated for warfarin monitoring in steady state, while the activated partial thromboplastin time (aPTT) was subsequently validated for heparin monitoring and for screening for hemophilia in affected families.^6^ Despite these tests being developed and validated for specific clinical indications, they are frequently ordered as routine screening tests in unselected patients, and often ordered together, which suggests a gap in physician understanding of coagulation, coagulation test limitations, and appropriate test usage.^6^, ^7^, ^8^ The excessive ordering of PT/aPTT testing is a particular concern, as the attempt to correct mild elevations of PT/aPTT could result in inappropriate transfusion of frozen plasma (FP).^9^, ^10^ FP is often transfused in patients with mild abnormalities in coagulation tests and corrects these values in only a minority of patients.^9^, ^11^, ^12^, ^13^, ^14^ A multicenter national audit in the United Kingdom identified that 41.0% of FP transfusions were administered to patients with a mildly prolonged INR and no bleeding.^12^ A single‐center Canadian audit of FP use identified 62.6% of FP transfusions were administered for an INR of 0.9 to 1.8.^13^ A more recent Canadian audit of FP transfusions at three large tertiary care centers identified that 81.5% of FP was transfused inappropriately, with 21.5% of these transfusions administered to patients with an INR of 1.5 to 1.7.^11^ The inappropriate transfusion of FP results in many negative outcomes, including the harm of transfusion reactions and increased costs to the health care system.^10^, ^11^ In addition to the specific harms of unnecessary coagulation testing, iatrogenic anemia secondary to frequent blood draws in the hospital is associated with increased risk of red blood cell transfusion.^4^, ^5^

Medical trainees (resident physicians and medical students) conduct a large proportion of laboratory test ordering at academic centers. Previous studies have demonstrated increased laboratory testing in teaching hospitals, compared to nonteaching hospitals, which may be associated with exposure to the training environment.^15^, ^16^, ^17^, ^18^ Despite the efforts of Choosing Wisely, in academic hospital medicine there is a culture that values overtesting, with a hidden curriculum that increased ordering of investigations demonstrates medical knowledge and thoroughness.^19^, ^20^ Increased laboratory test ordering among resident physicians has been linked to decreased knowledge regarding laboratory test appropriateness, test costs, and other harms of testing.^19^, ^20^, ^21^, ^22^ Trainees have also connected the ordering of laboratory tests to their own insecurities and fear of criticism from supervisors for not ordering laboratory tests.^23^ A lack of prioritization of teaching on appropriate use of testing and resource stewardship has previously been identified by trainees.^21^, ^23^

Coagulation has notoriously been a topic that medical trainees find challenging to learn, and the lack of understanding around coagulation and testing has led to widespread inappropriate ordering and misinterpretation of these tests.^7^, ^8^ We developed an online educational module on coagulation tests for trainees, with the goal of enhancing knowledge of appropriate inpatient coagulation laboratory test‐ordering practices.

## 2 METHODS

### 2.1 Root cause analysis and educational module development

We previously conducted a qualitative root cause analysis using a survey and focus group interviews with 10 internal medicine resident physicians at various levels of training at St. Michael's Hospital, a large academic center in Toronto, Canada, to explore gaps in their knowledge of coagulation testing and proposed solutions. Seventy percent of the surveyed trainees did not feel comfortable with their knowledge regarding coagulation and the appropriate use of coagulation tests. We developed the educational module in response to the following themes that emerged from the root cause analysis: (1) Trainees expressed discomfort with their knowledge regarding coagulation and attributed their suboptimal knowledge to the manner in which coagulation is traditionally taught; (2) traditional coagulation teaching often involves the use of Roman numerals and the emphasis on memorization of the coagulation cascade, which trainees expressed is difficult to grasp and not representative of in vivo physiology; (3) trainees identified a lack of connectedness between didactic coagulation teaching and clinical application; and (4) trainees identified a scarcity of practical resources on hemostasis and coagulation testing and expressed interest in a web‐accessible resource. In our extensive review of the currently available resources on the basics of coagulation and related testing, we did not come across any introductory, clinically relevant, interactive electronic educational modules on this topic targeted to trainees.

Our e‐module development team included hematology staff with expertise in coagulation and medical education. We created an educational module on coagulation testing targeting internal medicine trainees (including medical students and resident physicians) over a 6‐month period, with iterative improvements based on feedback from trainees and medical educators.

To evaluate the educational impact of the module, a 28‐question multiple choice pre‐ and postmodule quiz assessing knowledge of coagulation and appropriate use of coagulation testing was created and reviewed by hematology faculty with expertise in coagulation (Appendix S1). The quiz and the module underwent a validation process with regard to content, internal structure, response process, and relationship to other variables including level of training, with pilot testing and revisions.^24^, ^25^ The quiz had appropriate variability according to trainee level (including hematology and hemostasis fellows who participated in the validation process), and strong interitem reliability. The quiz was hosted on a web‐based platform (Survey Monkey: www.survey monkey.com).

Using Articulate 360 software^26^ the educational module was created and made available on a mobile‐friendly website platform at www.coagtesting.com. The content included in the educational module was based on the needs assessment at our center and included the basics of hemostasis, a simplified coagulation “cascade,” common inherited bleeding disorders, disseminated intravascular coagulation, and coagulopathy of liver disease. The educational module was tailored for trainees on a general internal medicine clinical rotation and reviewed the appropriate indications to order a PT/INR, aPTT, or both in this clinical setting, as well as the differential diagnosis for abnormalities in this testing.

### 2.2 Data collection

The study participants included resident physicians and medical students on a general internal medicine clinical rotation at our center. Participating trainees performed a premodule knowledge quiz on coagulation, and a subsequent postmodule knowledge quiz after completing the education module. To assess longer‐term knowledge retention, trainees were asked to repeat the knowledge quiz 3 to 6 months following their initial completion of the educational module. Trainees also provided responses regarding their subjective laboratory test‐ordering practices before and after completion of the module, using a 5‐point Likert scale.

The primary outcome of the study was to evaluate whether the educational module increases trainee knowledge on coagulation and appropriate coagulation test usage, based on postmodule quiz scores.

Secondary outcomes were to evaluate whether there was evidence of sustained knowledge retention on 3‐ to 6‐month postintervention quiz scores (latent quiz) and to determine whether the educational module resulted in a subjective change in trainee test‐ordering practices.

Mean and median quiz scores were collected, with participants’ responses described using a Wilcoxon signed‐rank test. A *P* value of<.05 was deemed to be statistically significant.

This study was approved by the St. Michael’s Hospital Research Ethics Board.

## 3 RESULTS

Fifty‐seven medical trainees on a general internal medicine clinical rotation during the time of our educational intervention were invited to participate in the study, and 50 trainees responded (88% response rate). Participation in the study was voluntary. The 50 participating trainees were 11 medical students and 39 internal medicine resident physicians (14 postgraduate year 1, 15 postgraduate year 2, and 10 postgraduate year 3). All trainees completed the pre‐ and postmodule quiz. Forty of the 50 trainees completed the 3‐ to 6‐month follow‐up quiz (latent quiz), a completion rate of 80%.

### 3.1 Knowledge quiz scores

**FIGURE 1:**
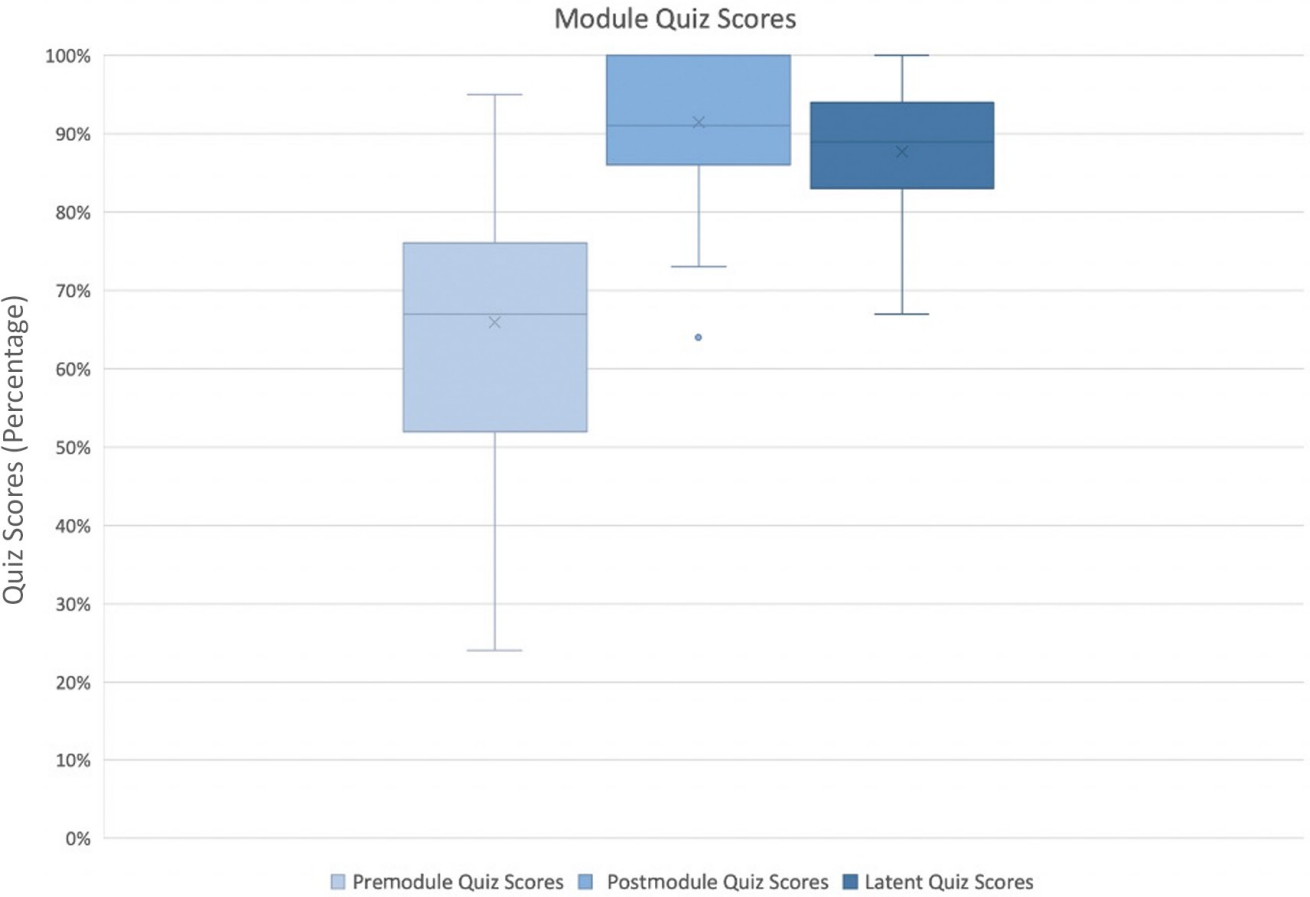
Premodule quiz, postmodule quiz, and latent quiz scores for all participants. The box plots demonstrate the interquartile range. The whiskers demonstrate the minimum and maximum scores for each quiz. Median quiz scores identified by horizontal lines, mean quiz scores identified by x and Quiz Scores (Percentage) identified by y

The median premodule quiz score was 67% (n = 50; range, 24%‐86%) with an increase of 24% to a median postmodule quiz score of 91% (n = 50; range, 64%‐100%; *P* < .001). There was evidence of sustained knowledge acquisition with a latent quiz median score of 89% (n = 40; range, 67%–100%; *P* < .001; Figure 1).

In the premodule quiz, only 6% of trainees correctly estimated the sensitivity and specificity of the PT and aPTT in detecting a bleeding disorder, with 94% of trainees overestimating the sensitivity and specificity of the aPTT in this setting. In the premodule quiz, only 32% of trainees correctly answered the question regarding the appropriate use of an inpatient aPTT test, and 48% of trainees correctly identified the cost of a PT test (Table 1).

**TABLE 1:** Percentage of questions correct on individual exams by topic

Question topic	Premodule quiz, % (n = 50)	Postmodule quiz, % (n = 50)	Latent quiz, % (n = 40)
Sensitivity and specificity of PT/aPTT testing	6	78	73
Appropriate use of inpatient PT/aPTT testing	32	94	85
Cost of PT/aPTT testing	48	90	75
Differential diagnosis of prolonged PT	54	96	98
Differential diagnosis of prolonged aPTT	82	98	98
Differential diagnosis of prolonged PT and aPTT	54	86	85
End point of PT/aPTT laboratory test	72	96	98
Anticoagulant reversal	86%	94%	85%
Impact of anticoagulation on PT/aPTT testing	68	76	70
Impact of hemophilia diagnosis on PT/aPTT testing	42	82	78
Bleeding disorder prevalence	64	96	98
Clinical features of disseminated intravascular coagulation	84	100	93

Abbreviations: aPTT, activated partial thromboplastin time; PT, prothrombin time.

Following completion of the module, 78% of trainees correctly identified the sensitivity and specificity of aPTT in detecting a bleeding disorder. In the postmodule quiz, 94% correctly answered the question regarding the appropriate use of an aPTT test in the inpatient setting, and 90% correctly identified the cost of a PT test (Table 1).

### 3.2 Subjective ordering practices

When surveyed about their ordering practices in the premodule quiz, 86% of trainees answered “Agree” or “Strongly Agree” on a 5‐point Likert scale when asked whether they are more likely to order a laboratory test if it is listed in a predesigned order set. Forty percent of trainees answered “Agree” or “Strongly Agree” when asked if they would feel uncomfortable not ordering a laboratory test if it is included in a standard predesigned order set.

Of the trainees that participated in the latent quiz, prior to completion of the module, only 32.5% selected “Agree” or “Strongly Agree” on a 5‐point Likert scale when asked whether they feel comfortable with their use of coagulation testing in the hospital and feel that they order these tests appropriately. Following completion of the module, 82.5% of trainees answered “Agree” or “Strongly Agree” when asked about their comfort with their appropriate use of coagulation testing. Trainees also expressed that the educational module had a positive impact on their learning. In addition, trainees expressed that they were more likely to consider the sensitivity, specificity, and cost of laboratory investigations before ordering them following completion of the educational module.

## 4 DISCUSSION

We demonstrated a significant increase in trainee coagulation knowledge after implementation of a targeted educational intervention. The median premodule quiz score was 67%, which increased to a median of 91% postmodule quiz score. In our study, we were also able to demonstrate sustained knowledge retention up to 6 months following completion of the educational module (latent quiz median score of 89%). Trainees also reported a subjective change to their test‐ordering practices following completion of our module, and that they would now be more likely to consider sensitivity, specificity, and cost before ordering a laboratory test.

While the PT and aPTT are often considered “routine” tests, they have specific indications, and the sensitivity and specificity of these tests for detecting a bleeding disorder is only 1% to 2%.^7^, ^27^ There are many harms associated with inappropriate coagulation testing, including increased risk of exposure to blood components and products, a cascade of additional investigations and referrals, delays to patient care, patient and physician anxiety, iatrogenic anemia, and additional, often unnecessary costs to the health care system.^3^, ^4^, ^5^, ^7^, ^9^, ^10^, ^11^ In a previous study conducted at our center in the emergency department, the reduction of routine use of PT/aPTT testing in unselected patients was associated with an estimated cost saving of US$56,000 per year.^28^

As medical trainees order a significant proportion of laboratory tests at academic centers, this is an important group to target in clinical quality improvement interventions.^15^, ^16^ Prior work has also highlighted the importance of involving medical trainees in quality improvement initiatives targeting ordering practices.^29^, ^30^ It has been recognized that a focus on resource stewardship is important in medical education, and trainees have expressed that educational interventions are a valuable tool in impacting their laboratory test–ordering practices.^19^, ^20^, ^23^ Educational interventions have been shown to enhance appropriate laboratory test–ordering among trainees.^29^, ^31^ One of the reasons that our intervention was impactful is the involvement of medical trainees at each stage of its development.

Our study revealed further insight into medical trainee laboratory test–ordering practices, with 86% of trainees indicating they are more likely to order a laboratory test if it is listed in a predesigned order set, and 40% of trainees responding that they would feel uncomfortable not ordering a laboratory test if it is included in a predesigned order set. Our findings are in line with previous studies on resident physician ordering practices, with fear of criticism from supervisors and impact of the training environment previously linked to overordering of laboratory tests by trainees. Prior work has demonstrated that trainees are less likely to consider laboratory test cost and patient discomfort before ordering tests.^32^ Our study highlights the importance of incorporating education for trainees on the cost and appropriate use of laboratory testing, as well as the implementation of systems‐level changes to standardized order sets, ensuring laboratory tests such as PT and aPTT are not listed inappropriately as “routine” tests on order sets.

One of the strengths of our study was the validation of the assessment tool: the module knowledge quiz. Another strength is that the development of the e‐module was based on a needs assessment and root‐cause analysis, and both the module and knowledge quizzes went through iterative revisions based on trainee and medical educator feedback. Another strength is that in addition to short‐term knowledge, we also assessed longer‐term knowledge retention following our educational intervention. We also assessed subjective ordering practices of trainees, and factors that affected their decision to order laboratory tests.

A limitation of our study is that it took place at a single center and included a small sample size, and thus may not be representative of trainee ordering practices at other hospital sites and in other geographic locations. In addition, while we were able to assess subjective trainee ordering practices, our study did not evaluate whether our intervention affected coagulation test usage at our hospital. At our center, laboratory tests ordered by trainees are processed under the name of the staff physician, and therefore we were unable to evaluate whether our intervention had an objective decrease on coagulation test–ordering among our participating medical trainees. Another possible limitation is that the pre‐ and postmodule quizzes contained the same questions, which could have improved trainee scores on the postmodule quiz. To mitigate this risk, the questions were always presented in a random order, and trainees were unable to keep quiz questions and were not provided with quiz answers. In addition, significant time had elapsed between the premodule quiz and the latent quiz that was taken 3 to 6 months later.

Aside from the results at our center, in the first year after launching the educational module online, it was accessed and completed by over 5000 unique visitors worldwide, with use in Canada, the United States, the United Kingdom, Australia, France, and Saudi Arabia according to data from the website host. Furthermore, we have been able to ascertain that several visitors to the website have revisited the module multiple times, with an average of 2.02 uses per unique visitor. We have received positive feedback from trainees on the e‐module’s content, usability, and positive impact on education.

Future directions will include targeted development of the e‐module based on trainee feedback to tailor the contents to the specific needs of medical and surgical specialties. For example, our immediate next step will be to create three additional modules: one on coagulation testing for the emergency setting targeting emergency medicine and intensive care trainees, one on coagulation testing in the perioperative setting for surgical and anesthesia trainees, and one on appropriate testing for the ambulatory setting for family medicine trainees. Given the unique learning objectives and clinical contexts of each of these specialties, these tailored modules will feature specialty‐specific interactive cases to maintain engagement and clinical relevance.

## 5 CONCLUSIONS

We have developed an online educational module for trainees on inpatient coagulation testing. We have demonstrated sustained knowledge retention regarding coagulation and appropriate coagulation test ordering, as well as a subjective change to trainee ordering habits following participation in our educational intervention. Furthermore, our educational intervention has been accessed by users across several countries. The extent to which individuals have been accessing our educational module internationally suggests to us that there is a need for such a web‐based, accessible resource to bridge the coagulation knowledge gap. In the context of the COVID‐19 pandemic, which has disrupted traditional medical education, there is a clear need for innovative, interactive, and remotely accessible e‐learning resources.

## RELATIONSHIP DISCLOSURE

All authors declare no conflicts of interest.

## AUTHOR CONTRIBUTIONS

NG wrote the initial draft of the manuscript. MT, RS, NG, and MS all provided critical revisions to the manuscript. All authors reviewed drafts of the manuscript and approved the final version for submission.

## Supporting information


Appendix S1
