# Supplementary material for: Coagulation test understanding and ordering by medical trainees: Novel teaching approach
Source: Res Pract Thromb Haemost. 2022 Jun 17;6(4):10.1002/rth2.12746. doi: 10.1002/rth2.12746 (PMC9204395; doi:10.1002/rth2.12746)
Supplement: Supplementary file 1 — Appendix S1 [file RTH2-6--s001.doc]

**Enhancing Coagulation Test Understanding and Ordering by Medical Trainees**

Educational Module Pre-Quiz

1. Which of the following could cause an isolated prolonged aPTT with a normal PT/INR? Select all that apply.
2. Deficiency in Factors 8, 9, or 11
3. Deficiency in Factor 7
4. Heparin therapy
5. Warfarin therapy
6. Which of the following is correct?
7. PT assesses the intrinsic pathway
8. PT assesses the extrinsic pathway
9. aPTT assesses the extrinsic pathway
10. Factors 11 and 12 are part of the extrinsic pathway
11. Which of the following clinical scenarios could cause an isolated prolonged PT with a normal aPTT?
12. Early liver disease
13. Factor 11 deficiency
14. Hemophilia A
15. All of the above
16. The aPTT should be considered on the inpatient General Internal Medicine ward in the following scenarios:
17. Argatroban monitoring
18. Enoxaparin monitoring
19. Dabigatran monitoring
20. Warfarin monitoring
21. None of the above
22. Which of the following represents the cost per PT/INR test in Canadian dollars?
23. $0.5-$1
24. $1-$3
25. $3-$7
26. $8-$10
27. What is the end-point of the PT and aPTT tests?
28. Time to clot stability
29. Time to activation of fibrinolysis
30. Time to fibrin clot formation
31. Time to formation of platelet plug
32. Causes of low fibrinogen include which of the following:
33. Bleeding after major trauma
34. Severe liver disease
35. Warfarin therapy
36. A and B
37. Excessive anticoagulation due to warfarin can be reversed by:
38. Vitamin K
39. Protamine sulfate
40. Prothrombin complex concentrate
41. A and C
42. Vitamin K dependent factors are:
43. Factors 2, 8, 9, 12, Protein C, Protein S
44. Factors 2, 7, 9, 10, Protein C, Protein S
45. Factors 8 and 9, Anti-Thrombin
46. Factors 8 and 9, Protein C & Protein S.
47. Which of the following could cause both a prolonged PT and aPTT?
48. DIC
49. Von Willebrand disease
50. Hemophilia
51. All of the above
52. To diagnose factor 8 deficiency versus factor 9 deficiency which test should be done:
53. PT
54. aPTT
55. PT and aPTT
56. Factor Activity Assay
57. Patients with hemophilia will have abnormal test results for:
58. aPTT only
59. PT only
60. aPTT and PT
61. PT, aPTT and platelet count
62. Which of the following could cause prolonged PT and aPTT?
63. Severe liver disease
64. DIC
65. Renal failure
66. A and B
67. Which of the following is true regarding the sensitivity and specificity of PT and aPTT for the detection of an underlying bleeding disorder:
68. The sensitivity and specificity is approximately 1-2%
69. The sensitivity and specificity is approximately 5-10%
70. The sensitivity and specificity is approximately 15-30%
71. The sensitivity and specificity is approximately 40-60%
72. Which of the following is the most prevalent bleeding disorder?
73. Hemophilia A and B
74. Von Willebrand Disease
75. Factor 11 Deficiency
76. Factor 12 Deficiency
77. Factor 5 Leiden

1. Which of the following is true regarding PT and aPTT testing and bleeding disorders?
2. Hemophilia can be detected by both PT and aPTT
3. Hemophilia can be detected by aPTT only
4. von Willebrand disease can be detected by PT only
5. Hemophilia and von Willebrand Disease cannot be detected by PT or aPTT
6. Which of the following is true?
7. Hemophilia is a disorder of secondary hemostasis
8. Hemophilia classically presents with mucocutaneous bleeding
9. Hemophilia is inherited in an autosomal recessive pattern
10. Hemophilia A is inherited factor 9 deficiency
11. Which of the following is true?
12. Primary hemostasis involves activation of coagulation factors
13. In primary hemostasis, tissue factor adheres platelets to exposed subendothelial collagen
14. Tissue factor is released from injured endothelial cells and helps to initiate coagulation
15. In secondary hemostasis, the intrinsic Xase directly activates thrombin, resulting in the formation of the insoluble fibrin clot
16. Which of the following is incorrect with regards to the impact of anticoagulants on PT and aPTT testing?
17. Warfarin therapy can cause a prolonged PT
18. Heparin therapy can cause a prolonged PT with a normal aPTT
19. Argatroban therapy can cause a prolonged aPTT and PT
20. Direct Xa inhibitors such as apixaban and rivaroxaban can cause a prolonged PT
21. Which of the following is correct?
22. All coagulation factors are formed by the liver except for factor 9
23. Coagulation factor 8 is carried by von Willebrand factor
24. Fibrinogen is formed by the liver
25. B and C
26. Which of the following is not typically associated with DIC?
27. Thrombocytosis
28. Prolonged PT
29. Prolonged aPTT
30. Thombocytopenia
31. Hypofibrinogenemia
32. Which of the following could result in a high INR in the General Internal Medicine inpatient setting?
33. Malnutrition
34. Antibiotic use
35. Renal failure
36. A and B
37. I am more likely to order a laboratory test if it is listed in an order set:
38. Strongly agree
39. Agree
40. Neither agree nor disagree
41. Disagree
42. Strongly disagree
43. I feel comfortable with my use of coagulation tests in hospital and feel that I order them appropriately:
44. Strongly agree
45. Agree
46. Neither agree nor disagree
47. Disagree
48. Strongly disagree
49. I feel comfortable with my understanding of the biology of hemostasis:
50. Strongly agree
51. Agree
52. Neither agree nor disagree
53. Disagree
54. Strongly disagree
55. I feel comfortable with my understanding of anticoagulants, including appropriate monitoring and use of reversal agents
56. Strongly agree
57. Agree
58. Neither agree nor disagree
59. Disagree
60. Strongly disagree
61. If a lab test is included in a pre-designed order set (for example a General Internal Medicine admission order set), I am more likely to order that test:
62. Strongly agree
63. Agree
64. Neither agree nor disagree
65. Disagree
66. Strongly disagree
67. If a lab test is included in a pre-designed order set (for example a General Internal Medicine admission order set), I would feel uncomfortable not ordering that test:
68. Strongly agree
69. Agree
70. Neither agree nor disagree
71. Disagree
72. Strongly disagree
